# Biochemical and Molecular Characterization of the Rice Chalcone Isomerase Family

**DOI:** 10.3390/plants10102064

**Published:** 2021-09-30

**Authors:** Sang-Il Park, Hye-Lin Park, Seong-Hee Bhoo, Sang-Won Lee, Man-Ho Cho

**Affiliations:** Department of Genetics and Biotechnology, Kyung Hee University, Yongin 17104, Korea; 2790park@naver.com (S.-I.P.); hlpark@khu.ac.kr (H.-L.P.); shbhoo@khu.ac.kr (S.-H.B.)

**Keywords:** chalcone isomerase, rice, OsCHI family, phytoalexin, sakuranetin

## Abstract

Chalcone isomerase (CHI) is a key enzyme in flavonoid biosynthesis. In plants, CHIs occur in multigene families, and they are divided into four types, types I–IV. Type I and II CHIs are bona fide CHIs with CHI activity, and type III and IV CHIs are non-catalytic members with different functions. Rice contains seven CHI family genes (*OsCHI*s). Molecular analysis suggested that OsCHI3 is a type I CHI, and the other OsCHIs were classified into types III and IV. To elucidate their biochemical functions, OsCHI1, OsCHI3, OsCHI6, and OsCHI7 were expressed in *Escherichia coli*, and the recombinant OsCHI proteins were purified. An activity assay of recombinant OsCHIs showed that OsCHI3 catalyzed the isomerization of naringenin chalcone and isoliquiritigenin, whereas the other recombinant OsCHIs had no CHI activity. OsCHI3 also exhibited a strong preference to naringenin chalcone compared to isoliquiritigenin, which agrees well with the catalytic properties of type I CHIs. These results ascertain OsCHI3 to be a bona fide CHI in rice. *OsCHI3* and the other *OsCHI*s were expressed constitutively throughout the rice growth period and different tissues. *OsCHI3* expression was induced immediately in response to ultra-violet (UV) stress, suggesting its involvement in the biosynthesis of sakuranetin, a flavonoid phytoalexin in rice.

## 1. Introduction

Flavonoids are a major group of plant secondary metabolites that play a role in pigmentation, development, and reproduction as well as participate in defense against biotic and abiotic stresses as UV-protectants, antioxidants, and antimicrobial agents [1,2,3]. They are also health-beneficial phytonutrients in the human diet, with diverse biological activities, including hepatoprotective, anti-inflammatory, antibacterial, and anticancer properties [4,5].

Flavonoid biosynthesis is branched from the general phenylpropanoid pathway [1,2]. Chalcone synthase (CHS) is the first committed enzyme for flavonoid biosynthesis and catalyzes the formation of chalcones from one *p*-coumaroyl-CoA and three malonyl-CoAs [6,7,8,9]. Chalcone isomerase (CHI) sequentially catalyzes the stereospecific isomerization of chalcones into the corresponding (2*S*)-flavanones, providing basic backbones for a wide array of flavonoids [10,11]. In plants, *CHI* genes exist in multigene families and are divided into four types, types I–IV [12,13,14,15]. Types I and II CHIs are bona fide CHIs, having catalytic activities, and type III and IV CHIs are non-catalytic members with different physiological functions in plants [11,12,13,16,17,18,19]. In most plant species, CHS catalyzes the formation of 4,2′,4′,6′-tetrahydroxychalcone (naringenin chalcone) from *p*-coumaroyl-CoA and malonyl-CoAs. Naringenin chalcone is then isomerized to naringenin (5,7,4′-trihydroxyflavanone) by type I CHI, providing a basic backbone for general flavonoids [12,15]. In legumes, 4,2′,4′-trihydroxychalcone (isoliquiritigenin) is formed by CHS in conjunction with chalcone reductase [20,21]. Type II CHIs then isomerize isoliquiritigenin to liquiritigenin (7,4′-dihyrroxyflavanone), leading to isoflavonoid biosynthesis [11,12,14]. Isoflavonoids play an important role in legumes as phytoalexins, antimicrobial secondary metabolites produced in plants in response to pathogen attacks [3].

Rice produces a diverse variety of diterpenoid and phenolic phytoalexins in response to pathogen infections and UV stress [9,22,23,24,25,26,27]. Sakuranetin is a well-known rice phytoalexin and is a 7-methylated form of the flavanone naringenin, which is isolated from UV-treated rice leaves and exhibits antimicrobial activity against rice blast fungus (*Magnaporthe grisea*) [23]. Further research demonstrated that sakuranetin also has antimicrobial activity against several fungal and bacterial pathogens in rice, such as brown spot fungus (*Bipolaris oryzae*), grain rot bacterium (*Burkholderia glumae*), and blight bacterium (*Xanthomonas oryzae* pv. *oryzae*) [23,26]. Moreover, phenylpropanoid pathway and *CHS* genes are upregulated by UV irradiation and participate in sakuranetin synthesis under UV stress [9,25,27].

The rice genome contains seven *CHI* family members (*OsCHI*s). To delineate their physiological roles, we carried out molecular and biochemical characterization of the *OsCHI* family. The CHI activity assay showed that OsCHI3 is the only catalytic CHI, and the other OsCHIs are non-catalytic. Such catalytic activity and phylogenetic relationships indicate that OsCHI3 is a bona fide type I CHI in rice. In addition, the UV-induced expression of *OsCHI3* suggested its involvement in sakuranetin biosynthesis. Expression patterns of six non-catalytic *OsCHI* family members in different tissues and developmental stages were examined, and their possible role was discussed.

## 2. Results

### 2.1. The CHI Family in Rice

A search of the MSU Rice Genome Annotation Project (RGAP) database [28] indicated that the rice *CHI* family is comprised of seven members designated as *OsCHI1*–*OsCHI7* (Table 1). The open reading frames (ORFs) and amino acids lengths of bona fide CHIs of *Arabidopsis*, alfalfa, pea, soybean, and maize are 657 to 741 nucleotides and 218 to 246 amino acids long, respectively [12,13,14,29]. Of the *OsCHIs*, *OsCHI1*, *OsCHI3*, *OsCHI5*, *OsCHI6*, and *OsCHI7* showed comparable ORF sizes (627–843 nucleotides) to bona fide *CHI*s, which encode polypeptides of 208–280 amino acids long (Table 1). The RGAP database suggests two splicing variants (Os03g60509.1 and Os03g60509.2) of *OsCHI3*. One variant (Os03g60509.1) has a slightly long ORF (888 nucleotides), and the other variant (Os03g60509.2) shows a similar ORF size to typical plant *CHI*s. Despite extensive attempts to clone *OsCHI3* cDNA from diverse rice tissues, only a smaller variant was cloned from rice leaves. These pieces of evidence suggest that the splicing variant Os03g60509.2 is an expressing form of *OsCHI3*. The ORF lengths of *OsCHI2* and *OsCHI4* are longer than those of other *CHI*s (Table 1) because of their large N-terminal extension (Figure S1).

**Figure 1:**
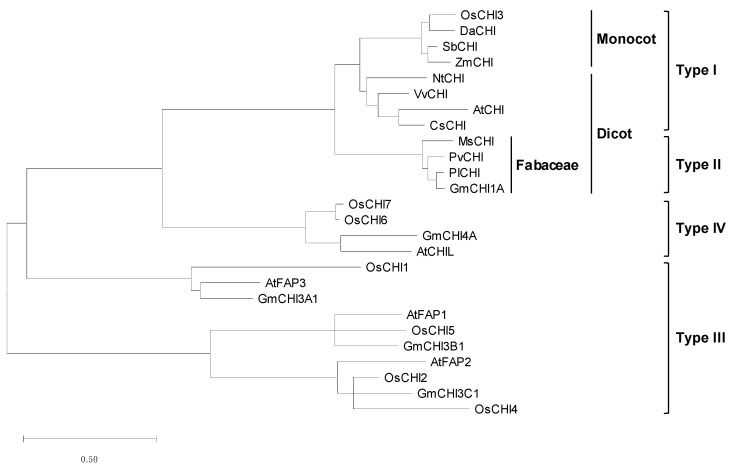
Phylogenetic analysis of OsCHIs and other plant CHIs. Amino acid sequences were aligned using Clustal W, and the phylogenetic tree was constructed by the maximum likelihood method using MEGA X. Scale bar denotes branch lengths measured in the number of substitutions per site. Amino acid sequences of plant CHIs used were DaCHI (CBX44252), ZmCHI (Q08704), SbCHI (XP_002463631), VvCHI (NP_001268033), AtCHI (P41088), NtCHI (NP_001312216), MsCHI (P28012), PlCHI (Q43056) GmCHI1A (NP_001235219), PvCHI (P14298), AtCHL (AY063786), AtFAP1 (Q9M1x2), AtFAP2 (Q84RK2), AtFAP3 (Q9C8L2), GmCHI3A1(NP_001238390), GmCHI3B1 (NP_001351383), GmCHI3C1 (Q43056), and GmCHI4A (AAT94362).

Amino acid sequence homology and phylogenetic relationships between OsCHIs and other plant CHIs were analyzed to classify the OsCHI family (Figure 1 and Table S1). Among the OsCHIs, OsCHI3 is highly homologous to bona fide CHIs, showing 44.0–77.8% sequence identity (Table S1). The other OsCHI members showed 6.0–30.4% identity with bona fide CHIs (Table S1). Phylogenetic analysis revealed that OsCHI3 is classified as type I CHI (Figure 1). Along with type II CHIs, type I CHIs are bona fide CHIs with catalytic activity [11,12,15]. Type II CHIs are homologous to each other, with more than 70% peptide sequence identity, whereas the identity between types I and II is about 50% [11]. Similarly, OsCHI3 showed 44.0–48.9% identity with leguminous type II CHIs, such as *Medicago sativa* CHI (MsCHI), *Phaseolus vulgaris* CHI (PvCHI), *Pueraria lobata* CHI (PlCHI), and *Glycine max* CHI1A (GmCHI1A). Within type I CHIs, OsCHI3 exhibited greater homology with monocot CHIs, such as *Deschampsia antarctica* CHI (DaCHI), *Zea mays* CHI (ZmCHI), and *Sorghum bicolor* CHI (SbCHI), with 77.8%, 73.6%, and 76.5% identities, respectively, than to dicot CHIs (51.4–58.9% identity), such as *Arabidopsis thaliana* CHI (AtCHI, also known as TT5), *Vitis vinifera* CHI (VvCHI), *Nicotiana tabacum* CHI (NtCHI), and *Citrus sinensis* CHI (CsCHI) (Figure 1 and Table S1). Phylogenetic analysis also showed that OsCHI3 forms a separate branch with other monocot type I CHIs (Figure 1). Phylogenetic analysis showed that there is no type II CHI in the rice CHI family.

OsCHI1, OsCHI2, OsCHI4, and OsCHI5 are grouped into the type III CHI subfamily (Figure 1). It has been suggested that type III CHIs are distributed widely in land plants and green algae [12,13]. Previous studies have shown that CHI is evolved from fatty acid-binding proteins (FAPs) [13,14]. In the *AtCHI* family, three members (*AtFAP1–3*) were found to encode FAPs, categorized as type III CHIs [13]. OsCHI1 is closely related to AtFAP3 and forms one subgroup in the type III CHI subfamily (Figure 1). AtFAP1 and AtFAP2 form the other subgroup, which includes OsCHI 2, OsCHI4, and OsCHI5 (Figure 1). The peptide length of AtFAP2 is longer than that of typical CHIs and is related closely to GmCHI3C1, OsCHI2, and OsCHI4 (Figure 1) [14]. The peptide lengths of these CHI members are about 400 amino acids long (Table 1) [14]. Unlike the type III CHIs, type IV CHIs are found only in land plants [12,13]. OsCHI6 and OsCHI7 are classified as type IV CHIs accompanied by AtCHI-like (AtCHIL) and GmCHI4A (Figure 1) [14].

### 2.2. Analysis of Conserved Residues within CHIs regarding Substrate-Binding and Catalysis

**Figure 2:**
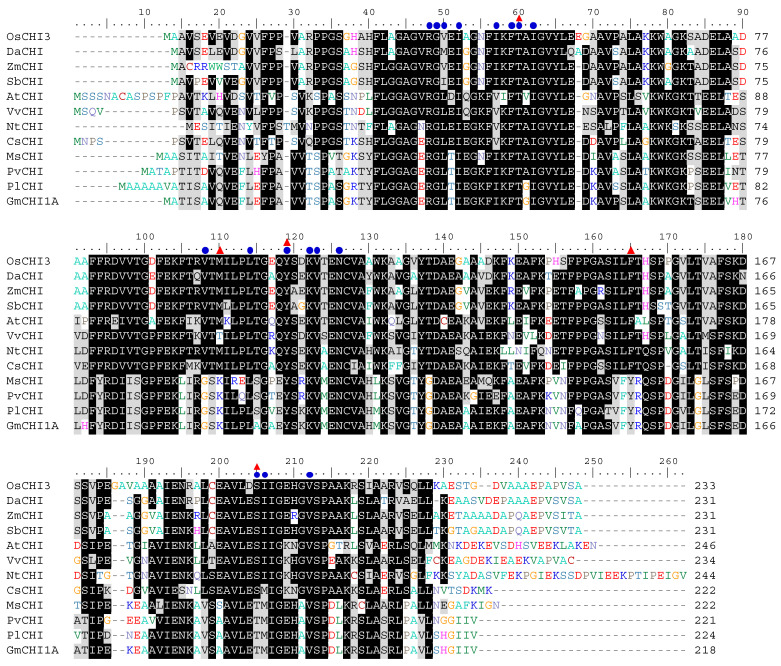
Multiple alignment of OsCHI3 with bona fide CHIs from other plant species. Identical and similar amino acid residues are shaded in black and grey, respectively. Blue circles above the residues indicate the conserved residues forming the substrate-binding cleft in MsCHI and DaCHI. Red triangles indicate the residues participating in the hydrogen-bond networks for catalysis.

Three-dimensional structures of MsCHI, AtCHI, and DaCHI were resolved by X-ray crystallography [13,29,30]. The crystal structures revealed the residues forming the substrate-binding clefts of the CHIs and the active site residues participating in the hydrogen-bond networks with substrates and water molecules for catalysis [29,30]. These substrate-binding and catalytic residues are well-conserved in both type I and type II CHIs (Figure 2) [11,12,14,29,30].

The substrate-binding cleft of type II MsCHI is largely apolar and consists of Arg 36, Gly 37, Leu 38, Phe 47, Thr 48, Ile 50, Tyr 106, Lys 109, Val 110, Asn 113, Thr 190, and Met 191 [29]. Most of these residues are conserved in type I DaCHI (Figure 2) [30]. In addition to these residues, Ile 38, Ile 43, Val 94, Met 96, and Leu 100 are involved in the formation of the substrate-binding cleft in DaCHI [30]. Among OsCHIs, the residues forming the substrate-binding cleft are conserved highly in OsCHI3, and they are largely variable in the other OsCHIs (Figure 2 and Figure S1). Type I CHIs show a strong preference for naringenin chalcone, whereas type II CHIs exhibit a broader substrate preference, catalyzing the isomerization of both naringenin chalcone and isoliquiritigenin [11,12,14,15]. The Thr 190 and Met 191 residues of MsCHI have been suggested to participate in substrate preference, and they are conserved among type II CHIs (Figure 2) [29]. These Thr and Met residues are substituted mostly to Ser and Ile, respectively, in type I CHIs, including OsCHI3 (Figure 2). The conserved Val residue (Val 94 in DaCHI) in type I CHIs is replaced with Gly or Arg in type II CHIs (Figure 2). Multiple sequence alignments of bona fide CHIs showed that Leu 38 in MsCHI is conserved in type II CHIs and dicot type I CHIs, whereas it is substituted with Met, Val, and Ile in monocot DaCHI, ZmCHI, and SbCHI, respectively (Figure 2). In OsCHI3, this residue is changed to Val, similar to ZmCHI (Figure 2). The conserved Ile residue (Ile 43 in DaCHI) in monocot type I and type II CHIs is substituted with Val in dicot type I CHIs (Figure 2).

In MsCHI, Thr 48, Lys 97, Tyr 106, Tyr 152, and Thr 190 are involved in the hydrogen-bond networks with substrates and water molecules [29,31]. Of these residues, Thr 48 and Tyr 106 are conserved in all bona fide CHIs (Figure 2) [29,31]. They form hydrogen bonds with catalytic water molecules [29,30,31]. Lys 97 is mostly substituted with Met, and Tyr 152 is changed to Phe in type I CHIs, including OsCHI3 and DaCHI (Figure 2) [31]. As mentioned above, Thr 190 is substituted to Ser in type I CHIs (Figure 2).

### 2.3. Cloning and Heterologous Expression of OsCHIs

To elucidate the biochemical functions of OsCHIs, we tried to clone *OsCHI* genes from rice tissues. The cDNAs of *OsCHI*s were cloned successfully from rice leaves. Regarding the production of recombinant OsCHI proteins, each *OsCHI* cDNA was inserted into the expression vector pET-28a. Heterologous expression of the N-terminal His-tagged OsCHI proteins in *Escherichia coli* BL21(DE3) and Rosetta 2(DE3) strains was attempted under various induction conditions. OsCHI3 was expressed successfully in *E. coli* Rosetta 2 cells as a soluble protein by 0.5 mM isopropyl β-D-thiogalactopyranoside (IPTG) at an 18 °C growth temperature (Figure S2). OsCHI6 and OsCHI7 were expressed as soluble forms in *E. coli* BL21 cells by 0.5 mM IPTG under an induction temperature of 18 °C (Figure S2). The heterologous expression of OsCHI1 was attempted in both *E. coli* strains, and only a small amount was produced in *E. coli* Rosetta 2 cells as a soluble protein at an 18 °C growth temperature following induction with 0.5 mM IPTG (Figure S2). OsCHI2, OsCHI4, and OsCHI5 were not expressed as a soluble form in both *E. coli* strains.

**Figure 3:**
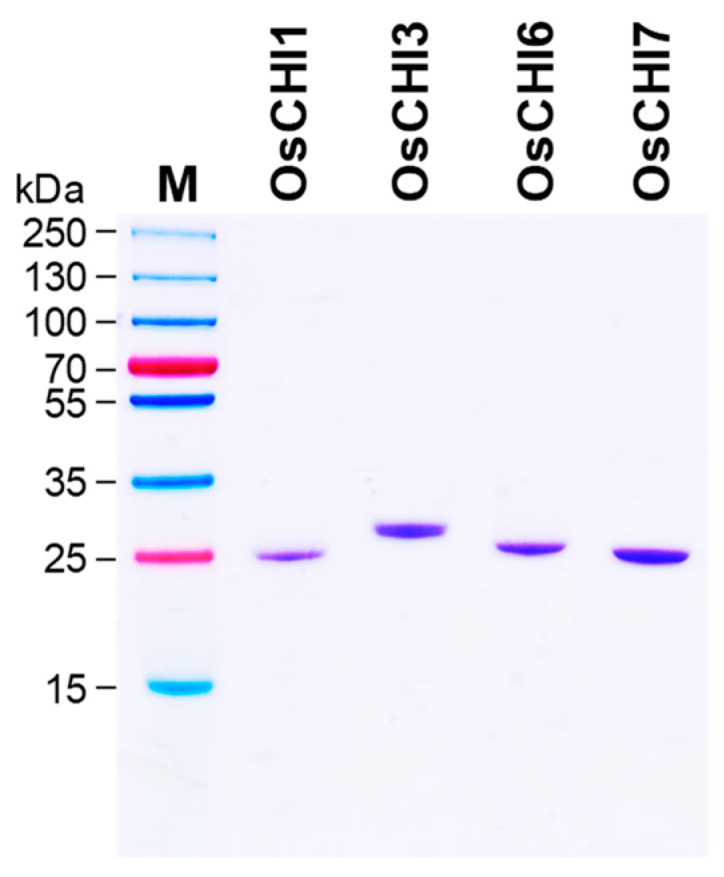
Purification of recombinant OsCHIs expressed in *E. coli*. The His-tagged recombinant OsCHI1, OsCHI3, OsCHI6, and OsCHI7 proteins were expressed as a soluble form in *E. coli*. The recombinant OsCHI3, OsCHI6, and OsCHI7 proteins were purified with Ni^2+^-affinity chromatography. OsCHI1 was purified with Ni^2+^-affinity chromatography followed by cation-exchange chromatography. M, Molecular weight marker.

Purification of recombinant OsCHI1, OsCHI3, OsCHI6, and OsCHI7 was attempted with Ni^2+^-affinity chromatography. OsCHI3, OsCHI6, and OsCHI7 were purified to apparent homogeneity by affinity chromatography, whereas the OsCHI1 fraction contained a large amount of non-specific proteins (Figure 3 and Figure S2). The affinity chromatography fraction was subjected to further cation-exchange chromatography to remove the unwanted proteins from the OsCHI1 preparation, leading to a successful purification of recombinant OsCHI1 to apparent homogeneity (Figure 3 and Figure S2). The purified OsCHI1, OsCHI3, OsCHI6, and OsCHI7 exhibited molecular masses of 25, 28, 25, and 25 kDa on SDS-PAGE, respectively, which are consistent with their theoretical molecular masses (Figure 3).

### 2.4. CHI Activity and Kinetic Properties of OsCHIs

Plant CHI families include bona fide CHIs and non-catalytic members [11,12,13,14,15]. The catalytic activity of recombinant OsCHI1, OsCHI3, OsCHI6, and OsCHI7 was examined with two substrates, naringenin chalcone and isoliquiritigenin. As expected, type I OsCHI3 showed CHI activity for naringenin chalcone, indicating that it is a bona fide CHI in rice (Figure S3), while the other recombinant OsCHIs showed no detectable CHI activity (Figure S4). As mentioned above, these OsCHIs have large substitutions in the conserved amino acid residues participating in the formation of their substrate-binding clefts and hydrogen bond networks (Figure S1), which can lead to loss of catalytic activity. Type I CHIs have long been thought to be a naringenin chalcone-specific enzyme [11,12,32]. A recent study showed that type I DaCHI also used isoliquiritigenin as a substrate, with a much lesser efficiency [30]. CHI activity for isoliquiritigenin was not detected under the same assay condition (0.1 µg of OsCHI3/mL) as for naringenin chalcone, and an enzyme reaction with a large amount (440 µg/mL) of OsCHI3 showed very slow isomerization of isoliquiritigenin to liquiritigenin (Figure S3).

The kinetic parameters of recombinant OsCHI3 towards naringenin chalcone and isoliquiritigenin were determined (Table 2). The *K*_M_ values of OsCHI3 for naringenin chalcone and isoliquiritigenin were 11.60 µM and 50.95 µM, respectively (Table 2). These values are comparable to those of other bona fide CHIs, ranging from 1 µM to 112 µM [10,11,12,30,32,33,34,35,36,37]. The *k*_cat_ values of OsCHI3 for the two examined substrates were very different, with 69.35 s^−1^ and 9.214 × 10^−5^ s^−1^ for naringenin chalcone and isoliquiritigenin, respectively (Table 2). The *k*_cat_/*K*_M_ value of OsCHI for naringenin chalcone was 5.978 × 10^6^ M^−1^ s^−1^ (Table 2). CHIs have been appeared to be perfect enzymes [10,11,12,30,33,34,35,36,37]. The *k*_cat_/*K*_M_ values of type I CHIs range from 4.62 × 10^5^ M^−1^ s^−1^ to 1.92 × 10^8^ M^−1^ s^−1^ [12,30,32,37]. The isomerization efficiency (*k*_cat_/*K*_M_ value of 1.809 M^−1^ s^−1^) of OsCHI3 for isoliquiritigenin was extremely lower than that for naringenin chalcone. The difference in catalytic efficiencies of OsCHI3 for naringenin and isoliquiritigenin was much bigger than that (about 1000-fold) of DaCHI [30].

### 2.5. Analysis of OsCHI Gene Expression

**Figure 4:**
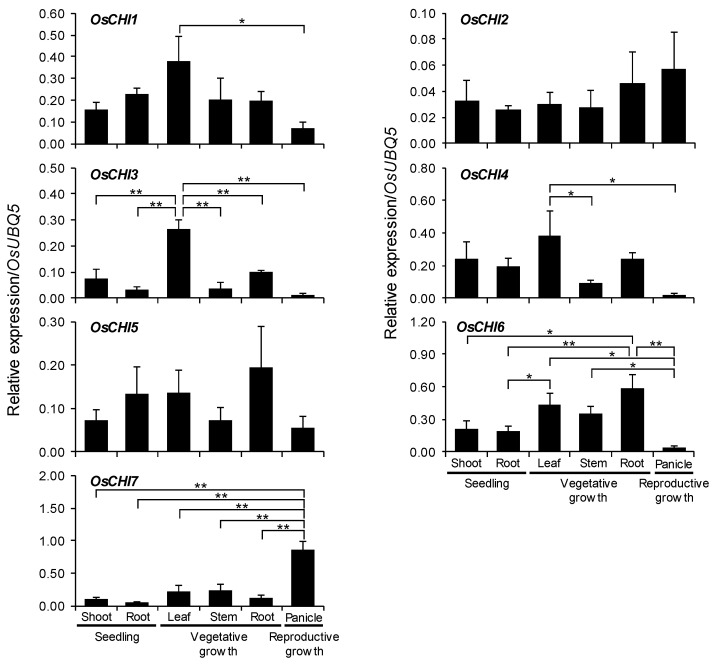
qRT-PCR analysis of *OsCHI* gene expression in rice seedlings and different tissues. A ubiquitin gene (*OsUBQ5*) was amplified using specific primers and was used as an internal control. Expression level of each *OsCHI* gene is presented as the relative expression compared to the *OsUBQ5* mRNA level. Asterisks indicate significant differences (* *p* < 0.05, ** *p* < 0.005, Tukey’s HSD-test). qRT-PCR analysis was performed on triplicate biological samples.

The spatial and temporal expression levels of *OsCHI*s in rice plants were examined through quantitative real-time polymerase chain reaction (qRT-PCR) analysis. The results showed that all *OsCHI*s are expressed constitutively throughout the growth period of rice plants (Figure 4 and Figure S5). At the seedling stage, the expression levels of each *OsCHI* were comparable in both shoots and roots (Figure 4 and Figure S5). At the vegetative growth stage, the bona fide *OsCHI3* was expressed highly in leaves compared to stems and roots, exhibiting the lowest expression level in stems (Figure 4 and Figure S5). *OsCHI4* expression was higher in leaves than in stems (Figure 4 and Figure S5). *OsCHI2*, *OsCHI6*, and *OsCHI7* were expressed similarly in leaves, stems, and roots in adult plants (Figure 4). In panicles, expression of *OsCHI3*, *OsCHI4*, and *OsCHI6* was very low compared to other tissues, while *OsCHI7* showed much higher expression, with about 4-fold higher expression than those in leaves and stems (Figure 4 and Figure S5).

**Figure 5:**
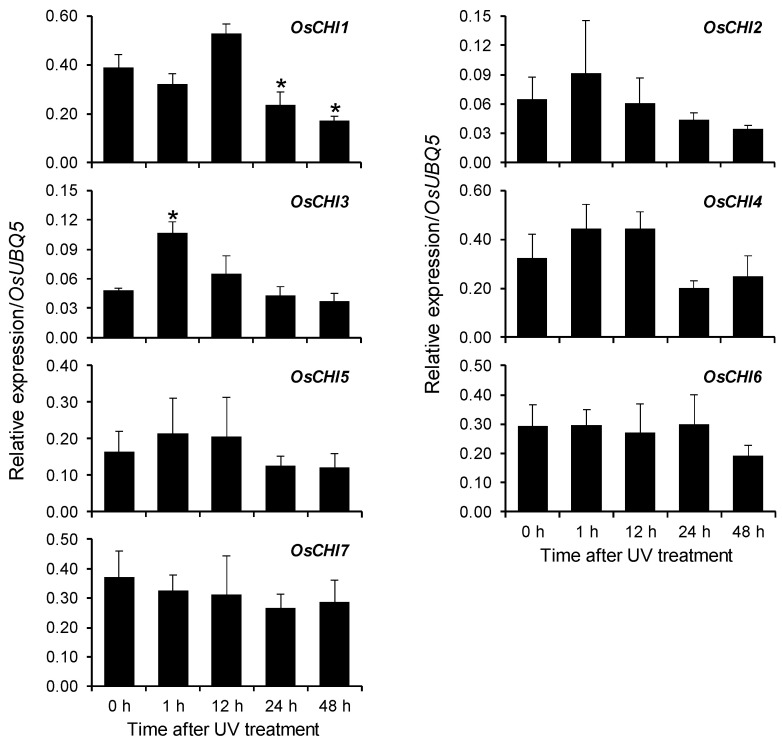
Expression of *OsCHI*s in rice leaves in response to UV treatment was analyzed using qRT-PCR. Rice plants treated with UV irradiation were collected at the designated time points and used to examine the expression of *OsCHI*s. A ubiquitin gene (*OsUBQ5*) was amplified using specific primers and used as an internal control. The expression level of each *OsCHI* gene was presented as the relative expression compared to the *OsUBQ5* mRNA level. Asterisks indicate significant differences (*p* < 0.05, *t*-test) between UV-untreated and UV-treated samples. qRT-PCR analysis was performed on the triplicated biological samples.

Previous studies have shown that UV stress stimulates the biosynthesis of the flavonoid phytoalexin sakuranetin in rice leaves [9,23,25,26]. High performance liquid chromatography (HPLC) analysis also showed the accumulation of sakuranetin in UV-treated rice leaves (Figure S6). Prior to sakuranetin accumulation, the expression of phenylpropanoid and flavonoid pathway genes, including *CHS* was upregulated in rice leaves in response to UV irradiation [9,25,27]. The expression of *OsCHI3* was increased immediately by UV irradiation, with the highest level at 1 h after UV treatment (Figure 5 and Figure S7). This UV-stimulated expression pattern is similar to that of *OsCHS8*, which encode a functional rice CHS isozyme [9]. The other *OsCHI*s showed no significant change in expression in response to UV treatment (Figure 5 and Figure S7).

## 3. Discussion

### 3.1. OsCHI3 Is a Bona Fide CHI in the OsCHI Family

In plants, the *CHI* families consist of several genes [13,14,38]. Soybean contains 12 *CHI* members [14], and five genes comprise the *CHI* family in *Arabidopsis* [13]. Eleven *CHI* family genes were identified from *Dracaena cambodiana* [38]. Likewise, seven *OsCHI* genes were found in the rice genome and were classified into three types, types I, III, and IV (Table 1 and Figure 1). Among family members, a few genes encode bona fide type I and type II CHIs [12,14,38]. In *Arabidopsis,* only one of five CHI members appears to be a bona fide type I CHI [13,39]. Soybeans have one type I CHI and three type II CHIs among 12 CHI family members [12,14]. Type I CHIs are ubiquitous in vascular plants and participate in naringenin formation, leading to flavonoid biosynthesis [11,12,14]. Type II CHIs have been thought to be legume specific and participate in biosynthesis of isoflavonoids [11,12,14]. Recent studies demonstrated that type II CHIs also exist in basal land plants, such as liverworts, *Selaginella moellendorffii*, and ferns [36,37]. Phylogenetic analysis predicted that the OsCHI family contains only one type I CHI, OsCHI3 (Figure 1). The *OsCHI3* gene was isolated previously from a bacterial artificial chromosome library of rice cv. Nipponbare based on sequence homology to *ZmCHI*, which was found to complement the *tt5* mutation of *Arabidopsis* [40,41]. An insertion mutation in the 5′-untranslated region of *OsCHI3* was reported to exhibit a reddish-brown pigmentation in rice hulls and internodes [42]. With the strict conservation of amino acid residues being important in substrate binding, recombinant OsCHI3 showed CHI activity to naringenin chalcone and isoliquiritigenin (Figure 2 and Table 2). Recombinant OsCHI1, OsCHI6, and OsCHI7 showed no detectable CHI activity (Figure S4), which agrees well with the non-catalytic nature of type III and IV CHIs [11,12,13,14,15]. Kinetic study showed that the *k*_cat_/*K*_M_ value of OsCHI3 for naringenin chalcone is comparable to that of other catalytic CHIs [12,30,32,37]. The catalytic efficiency of OsCHI3 to isoliquiritigenin is extremely lower than that to naringenin chalcone, which agrees well with the property of type I CHIs. These findings ascertain that OsCHI3 is a bona fide CHI in rice.

### 3.2. Monocot Bona Fide CHIs Categorized into a Separate Branch within Type I CHIs

It has been suggested that bona fide CHIs have strong homology, with more than 70% peptide sequence identity within the types (Table S1) [11]. Monocot type I CHIs, OsCHI3, DaCHI, ZmCHI, and SbCHI, were strongly homologous to each other with 73.6–88.3% identities (Table S1). However, the sequence identities between monocot and dicot type I CHIs were only 51.4–61.6% (Table S1), which resulted in these two groups being categorized into separate branches within type I CHIs (Figure 1). Monocot and dicot type I CHIs also showed a few mismatches in the residues forming the substrate-binding cleft. Leu 38 in MsCHI is conserved in type II CHIs and dicot type I CHIs, whereas it is substituted with other hydrophobic residues, such as Met, Val, and Ile, in monocot OsCHI3, DaCHI, ZmCHI, and SbCHI (Figure 2). This substitution of the Leu residue (Leu 38 in MsCHI) with other nonpolar residues is likely a characteristic change in monocot type I CHIs. The conserved Ile residue (Ile 43 in DaCHI) in monocot type I and type II CHIs is substituted to Val in dicot type I CHIs (Figure 2). These changes likely contributed to the subdivision of type I CHIs into monocot and dicot groups (Figure 1).

### 3.3. OsCHI3 Participates in UV-Induced Sakuranetin Synthesis in Rice Leaves

Pathogen infections and UV stress stimulate the production of phenolic phytoalexins, including sakuranetin, in rice [25,26,43]. Under UV stress, phenylpropanoid pathway genes have been shown to be upregulated prior to accumulation of phenolic phytoalexins in rice leaves [9,25,27]. A previous study demonstrated that rice contains two catalytic CHS isozymes, OsCHS8 and OsCHS24 [9]. Expression of both *OsCHS8* and *OsCHS24* appeared to be upregulated by UV irradiation, and they were shown to be redundantly involved in UV-induced sakuranetin synthesis in rice leaves [9]. Of these *OsCHS*s, *OsCHS8* expression was reported to be immediately induced by UV irradiation and subsequently decrease to non-UV-treated levels [9]. Likewise, *OsCHI3* is a bona fide CHI, and its expression was increased by UV irradiation, reaching its peak at 1 h after UV treatment (Figure 5 and Figure S7). UV-stimulated expression of the only catalytic OsCHI3 suggests that it contributes to UV-induced sakuranetin synthesis in rice leaves.

### 3.4. Prospective Role of Non-Catalytic OsCHI Members

Type III and IV CHIs are non-catalytic members of the CHI families, and their physiological functions have long been elusive. Of the *AtCHI* family members, three type III *CHI* genes were shown to encode FAPs [13]. A recent study showed that loss-of-function mutations in the *AtCHIL* gene encoding type IV CHI led to strong reduction of proanthocyanin and flavonol levels in seeds, suggesting that it is an enhancer of the flavonoid pathway [17]. Similarly, mutations in the *enhancer of flavonoid production* gene encoding a type IV CHI protein resulted in reduction of flavonols and anthocyanins in the flower petals of *Ipomoea nil* [16]. *Humulus lupulus* CHI-like 2, a type IV CHI, increased the level of demethylxanthohumol (DMX), a prenylated chalcone, in the engineered yeast harboring all genes required for DMX synthesis [18]. Several studies demonstrated that Type IV CHIs enhance flavonoid production through physical interactions with CHSs [18,19,37].

Constitutive expression of types III and IV OsCHIs throughout growth periods and tissues (Figure 4 and Figure S5) implies that they play a role in rice. However, little is known about the role of types III and IV OsCHIs. Very recently, transgenic rice plants expressing type III *OsCHI4* under the control of a stress-inducible promoter were reported to show improved tolerance to abiotic stresses, such as drought, salinity, and cold [44]. Proteomic analysis showed that type IV OsCHI6 and other related enzymes in phytoalexin biosynthesis were involved in the interaction between rice and *X. oryzae* pv. *Oryzae* [45]. qRT-PCR analysis showed that type IV *OsCHI7* was expressed highly in panicles compared to other rice tissues (Figure 4 and Figure S5). It has been known that CHI participates in seed coloration. The *tt5* mutation of *Arabidopsis* led to produce yellow seeds because of no accumulation of flavonoid pigments [39]. The proanthocyanin levels in seeds were remarkably decreased in the *atchil* mutants [17]. In this regard, the strong expression of *OsCHI7* in panicles suggests its possible involvement in the coloration of rice grains. Therefore, the physiological role of type III and IV OsCHIs, especially their stress-protective roles, need to be elucidated.

## 4. Materials and Methods

### 4.1. Plant Growth, UV Treatment, HPLC Analysis, and Materials

Wild-type rice (*Oryza sativa* L. subsp. *Japonica* cv. *Dongjin*) seeds were sterilized with 50% bleach for 30 min and germinated on Murachige and Skoog medium (Duchefa Biochemie, Haarlem, The Netherlands) in a growth chamber with a 16 h light/8 h dark photoperiod at 28 °C for two weeks. The rice seedlings were transferred to soil and grown in a greenhouse at 28 °C during the day. Shoot and root samples were collected from two-week-old rice seedlings. Leaves, stems, and roots were obtained from eight-week-old rice plants during the vegetative growth period. Panicles were harvested from 14-week-old rice plants. Eight-week-old rice plants were irradiated with UV light to examine the UV stress response. UV treatment and HPLC analysis were carried out according to the methods described by Park et al. [25].

Naringenin chalcone and isoliquiritigenin were purchased from Sigma-Aldrich (St. Louis, MO, USA). *E. coli* BL21(DE3) and Rosetta *2*(DE3) strains were obtained from Thermo Fisher Scientific (Waltham, MA, USA). Ni-NTA Agarose beads and CM Sepharose Fast Flow resin were purchased from Qiagen (Hilden, Germany) and Cytiva (Marlborough, MA, USA), respectively. Restriction enzymes were bought from New England Biolabs (Ipswich, MA, USA) and Enzynomics (Daejeon, Korea). Reagents for buffers and media were obtained from Sigma-Aldrich and Duchefa Biochemie.

### 4.2. Multiple Sequence Alignment and Phylogenetic Analysis of CHIs

Amino acid sequences of OsCHIs and other plant CHIs were obtained from the MSU RGAP database (http://rice.plantbiology.msu.edu/ accessed on 9 May 2021) and the National Center for Biotechnological Information (https://www.ncbi.nlm.nih.gov/ accessed on 28 April 2021) database, respectively. Multiple alignment of the amino acid sequences was conducted with Clustal W [46]. Phylogenetic analysis was performed with the maximum likelihood method, and Whelan and Goldman model using MEGA X [47,48]. Reliability of phylogeny was tested by the bootstrap method, with 100 of bootstrap replications.

### 4.3. Cloning of OsCHIs

Total RNA was extracted from eight-week-old wild-type rice leaves with RNAiso (Takara, Shiga, Japan). The first-strand cDNA was synthesized from the total RNA according to the methods described by Park et al. [9]. *OsCHI*s were amplified individually through PCR from the first-strand cDNA. The primer sets used for cloning of *OsCHI*s and PCR conditions are summarized in Table S2. The amplified PCR products were subcloned into the pJET1.2/blunt vector (Thermo Fisher Scientific). After sequence confirmation, each *OsCHI* was cut with suitable restriction enzymes, and inserted into the pET-28a(+) vector (Novagen, Madison, WI, USA). The resulting *OsCHI*/pET-28a(+) constructs were transformed into *E. coli* BL21(DE3) and Rosetta *2*(DE3) strains for the *OsCHI* expression.

### 4.4. Production and Purification of Recombinant OsCHIs

The *E. coli* transformants bearing each *OsCHI*/pET-28a(+) construct were grown in LB medium supplemented with the appropriate antibiotics at 37 °C. When an OD_600_ of ~0.6 was reached, different concentrations of IPTG (0.1–1 mM) were added into the cell culture to induce the production of OsCHI proteins, followed by an additional growth period of 16–18 h at various temperatures ranging from 16–37 °C. After induction, the cells were harvested by centrifugation (5000× *g* for 15 min), and the resulting cell pellets were resuspended with phosphate-buffered saline (10 mM Na_2_HPO_4_, 2 mM KH_2_PO_4_, 137 mM NaCl, 2.7 mM KCl,) supplemented with lysozyme (1 mg/mL) and phenylmethylsulfonyl fluoride (1 mM). The resuspended cells were lysed by sonication on ice, and the cell debris was removed by centrifugation at 15,000× *g* for 15 min. Recombinant OsCHI proteins were purified from the crude extract with Ni-NTA agarose chromatography according to the methods described by Park et al. [9]. The recombinant OsCHIs were eluted with 20–150 mM imidazole in Tris buffer (50 mM Tris, pH 8.0, 300 mM NaCl). The affinity chromatography fractions of recombinant OsCHI1 were applied to the CM-Sepharose column equilibrated with sodium phosphate buffer (50 mM, pH 7.0). The recombinant OsCHI1 protein was eluted with 200–300 mM NaCl in the equilibration buffer. The purified OsCHI proteins were analyzed with SDS-PAGE.

### 4.5. CHI Activity Assay

CHI activity was assayed by monitoring the decrease in absorbance at 390 nm (A_390_), which results from the conversion of chalcones to their corresponding flavanone [10]. A standard reaction mixture contained 50 µM of naringenin chalcone or isoliquiritigenin and the purified recombinant OsCHI protein (0.1 µg for OsCHI3, 100 µg for OsCHI1, and 500 µg for OsCHI6 and OsCHI7) in Tris buffer (50 mM, pH 7.5) to a total volume of 1 mL. Decreases in A_390_ were monitored using a Cary 300 Bio UV/Vis spectrophotometer (Varian, Mulgrave, Victoria, Australia). Due to spontaneous isomerization of substrates, the CHI activities of OsCHIs were determined by subtraction of un-catalyzed reactions from the OsCHI-catalyzed reactions. In the determination of kinetic parameters, the amounts of OsCHI3 protein used were 0.1 µg for naringenin chalcone and 500 µg for isoliquiritigenin, and substrate concentrations were 1–50 µM. Enzyme assays were performed in triplicate.

### 4.6. qRT- PCR Analysis of OsCHIs

Total RNAs from different rice tissues and UV-treated rice leaves were extracted using the Total RNA Prep Kit (Biofact, Daejeon, Korea), and cDNA was synthesized using SuPrimeScript RT premix, and OligodT (GeNet Bio, Daejeon, Korea). qRT-PCR was performed using Prime Q-Mastermix (GeNet Bio, Daejeon, Korea) on a Rotor-Gene Q real-time PCR cycler with Q-Rex Software (Qiagen, Hilden, Germany). Transcript levels were normalized to that of rice *ubiquitin 5* (*OsUBQ5*, Os01g22490) and rice *ubiquitin 1* (Os*UBQ1*, Os03g13170) transcripts as controls. The ΔCt method was applied to calculate OsCHI expression levels, and the primer specificity was assessed with a single peak in the melting curve. The primer sequences and annealing temperature for qRT-PCR are listed in Table S3. Gene expression analysis was performed on triplicated biological samples.

### 4.7. Statistical Analysis

Data are presented as mean ± standard deviation of three independent experiments. Multiple comparison of *OsCHI* expressions in rice seedlings and different tissues were performed with one-way ANOVA and Tukey’s HSD test. Significant differences of *OsCHI3* expression between UV-treated and untreated samples were determined by *t*-test. A *p*-value of <0.05 was considered as statistically significant.

## Figures and Tables

**Table 1:** Rice chalcone isomerase (*CHI*) gene family.

Name	Locus ID	Gene Description in the RGAP Database	ORF (bp) ^1^	Protein Size (aa ^2^)	CHI Type
OsCHI1	Os02g21520	chalcone isomerase 3, putative	843	280	III
OsCHI2	Os02g53810	expressed protein	1284	427	III
OsCHI3	Os03g60509	expressed protein	702	233	I
OsCHI4	Os06g10210	expressed protein	1290	429	III
OsCHI5	Os07g38390	chalcone isomerase, putative	792	263	III
OsCHI6	Os11g02440	chalcone-flavonone isomerase, putative	651	216	IV
OsCHI7	Os12g02370	chalcone-flavonone isomerase, putative	627	208	IV

^1^ ORF; Open reading frame, ^2^ aa; amino acid.

**Table 2:** Kinetic parameters of recombinant OsCHI3 ^1^.

Substrate	*K*_M_ (µM)	*V*_max_ (nmol s^−1^ mg^−1^)	*k*_cat_ (s^−1^)	*k*_cat_/*K*_M_ (M^−1^ s^−1^)
Naringenin chalcone	11.60 ± 0.78	2662 ± 207.9	69.35	5.978 × 10^6^
Isoliquiritigenin	50.95 ± 4.83	3.54 × 10^−3^ ± 1.25 × 10^−4^	9.214 × 10^−5^	1.809

^1^ The results represent the mean ± standard deviation of three independent experiments.

## Data Availability

The data presented in this study are available within this article.

## Supplementary Materials

The following are available online at https://www.mdpi.com/article/10.3390/plants10102064/s1, Figure S1: Multiple alignment of OsCHIs with other CHIs, Figure S2: Purification of recombinant OsCHSs expressed in *E. coli*, Figure S3: CHI activity assay of recombinant OsCHI3, Figure S4: CHI activity assay of recombinant OsCHI1, OsCHI6, and OsCHI7, Figure S5: qRT-PCR analysis of *OsCHI* gene expression in rice seedlings and different tissues, Figure S6: HPLC analysis of UV-treated (a) and untreated (b) rice leaves, Figure S7: Expression of *OsCHI*s in rice leaves in response to UV treatment was analyzed using qRT-PCR, Table S1: Amino acid homology of OsCHIs and bona fide CHIs, Table S2: Primer sequences and PCR conditions for *OsCHI* cloning, Table S3: Primer sequences and PCR conditions for quantitative real-time PCR analysis.
