# Supplementary material for: Biochemical and Molecular Characterization of the Rice Chalcone Isomerase Family
Source: Plants (Basel). 2021 Sep 30;10(10):2064. doi: 10.3390/plants10102064 (PMC8540780; doi:10.3390/plants10102064)
Supplement: Supplementary file 1 [file plants-10-02064-s001.zip › plants-1376743-supplementary.pptx]

## Slide 1
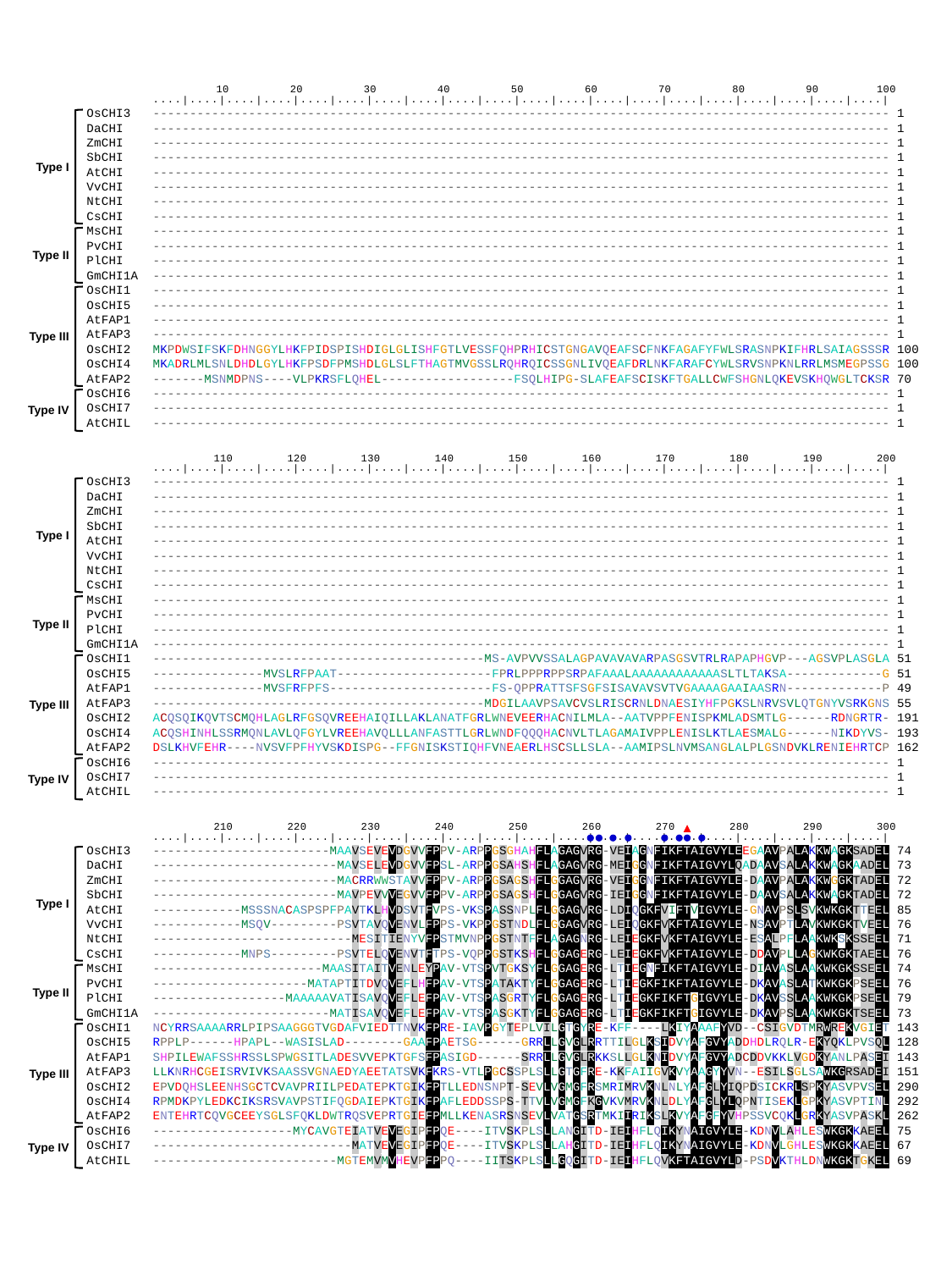

Type I
Type II
Type III
Type IV
Type I
Type II
Type III
Type IV
.
Type I
Type II
Type III
Type IV

## Slide 2
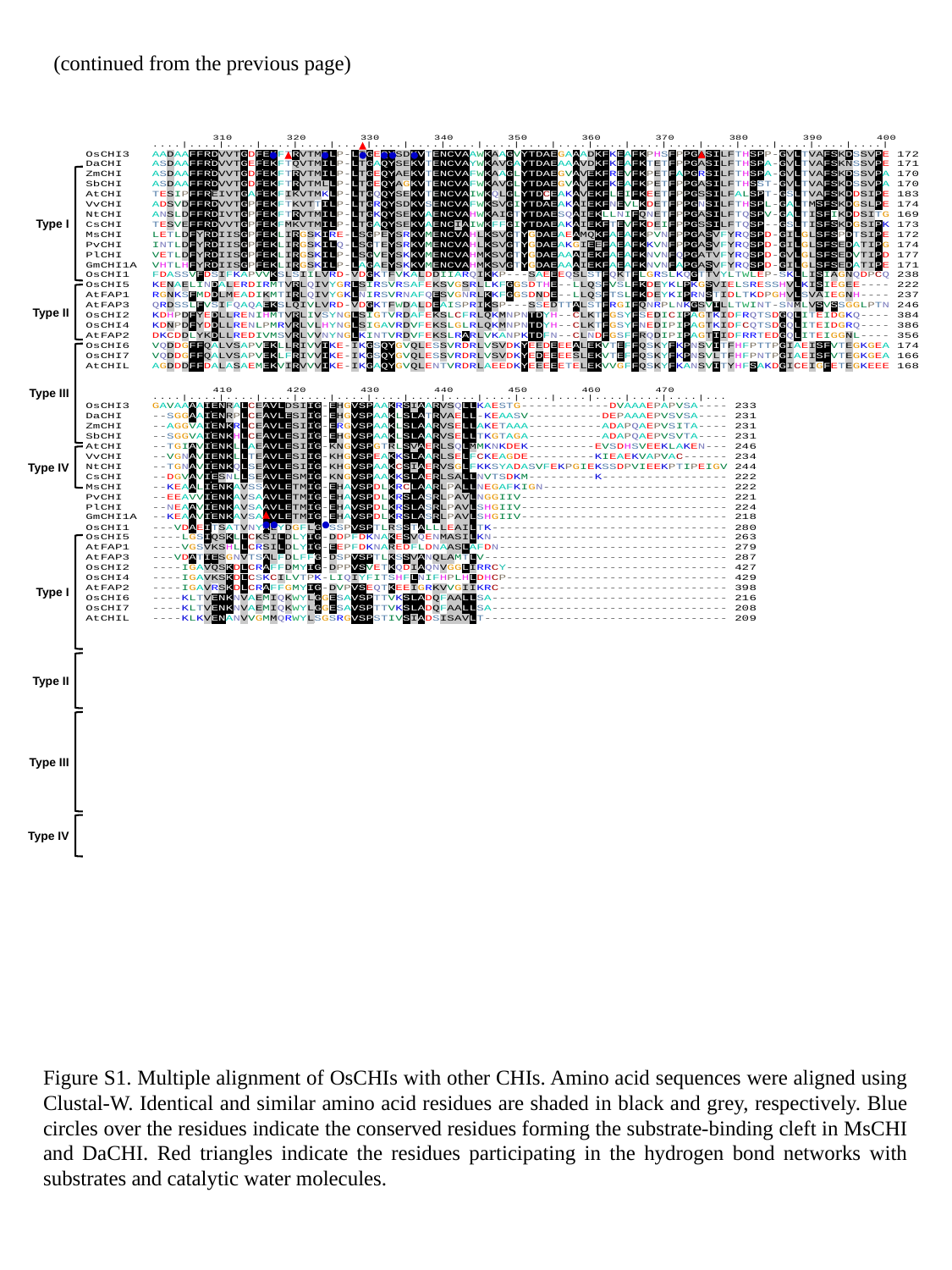

(continued from the previous page)
Type I
Type II
Type III
Type IV
Type I
Type II
Type III
Type IV
Figure S1. Multiple alignment of OsCHIs with other CHIs. Amino acid sequences were aligned using Clustal-W. Identical and similar amino acid residues are shaded in black and grey, respectively. Blue circles over the residues indicate the conserved residues forming the substrate-binding cleft in MsCHI and DaCHI. Red triangles indicate the residues participating in the hydrogen bond networks with substrates and catalytic water molecules.

## Slide 3
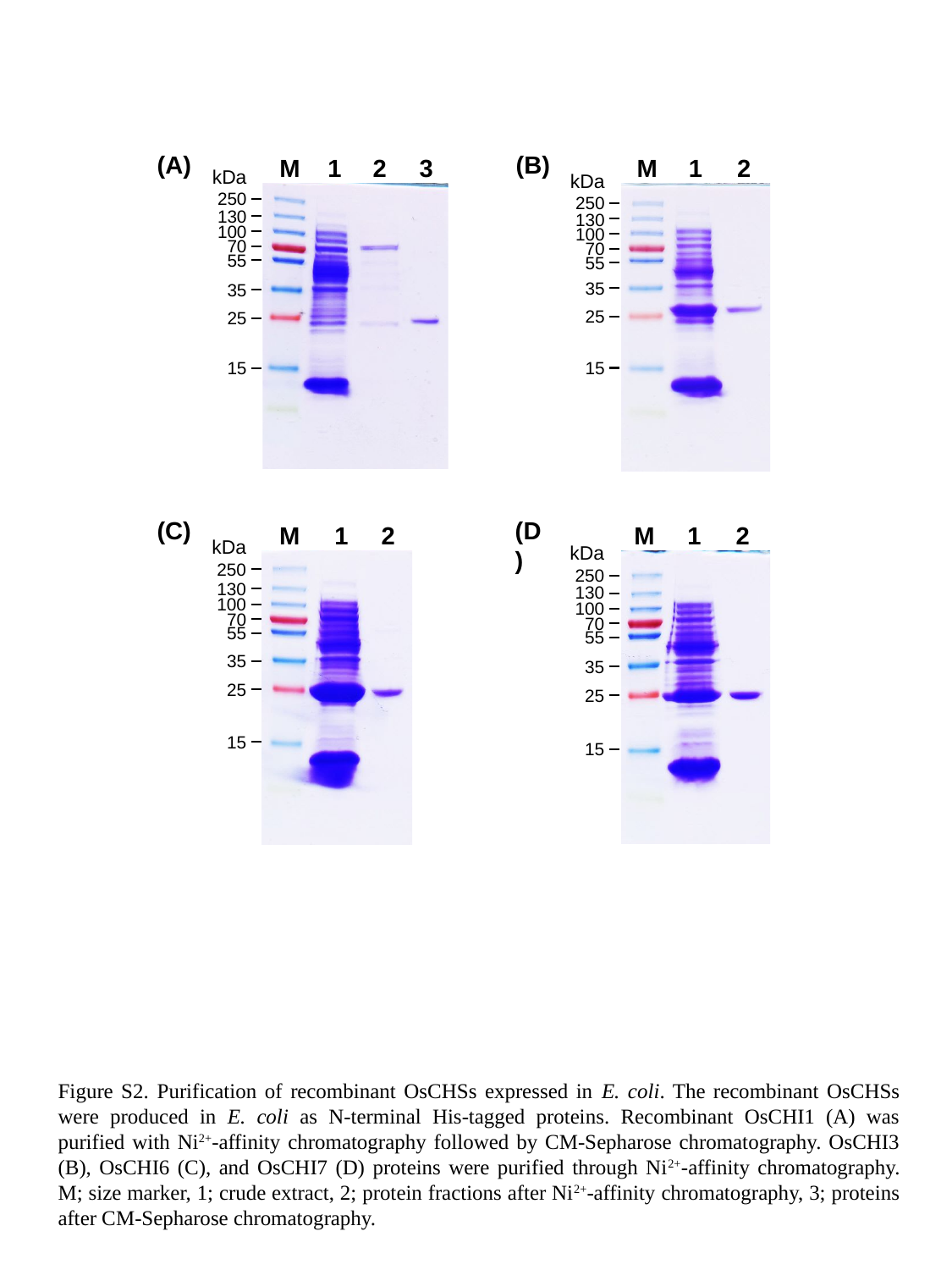

(B)
(A)
M 1 2 3
M 1 2
kDa
kDa
250
250
130
130
100
100
70
70
55
55
35
35
25
25
15
15
(C)
(D)
M 1 2
M 1 2
kDa
kDa
250
250
130
130
100
100
70
70
55
55
35
35
25
25
15
15
Figure S2. Purification of recombinant OsCHSs expressed in E. coli. The recombinant OsCHSs were produced in E. coli as N-terminal His-tagged proteins. Recombinant OsCHI1 (A) was purified with Ni2+-affinity chromatography followed by CM-Sepharose chromatography. OsCHI3 (B), OsCHI6 (C), and OsCHI7 (D) proteins were purified through Ni2+-affinity chromatography. M; size marker, 1; crude extract, 2; protein fractions after Ni2+-affinity chromatography, 3; proteins after CM-Sepharose chromatography.

## Slide 4
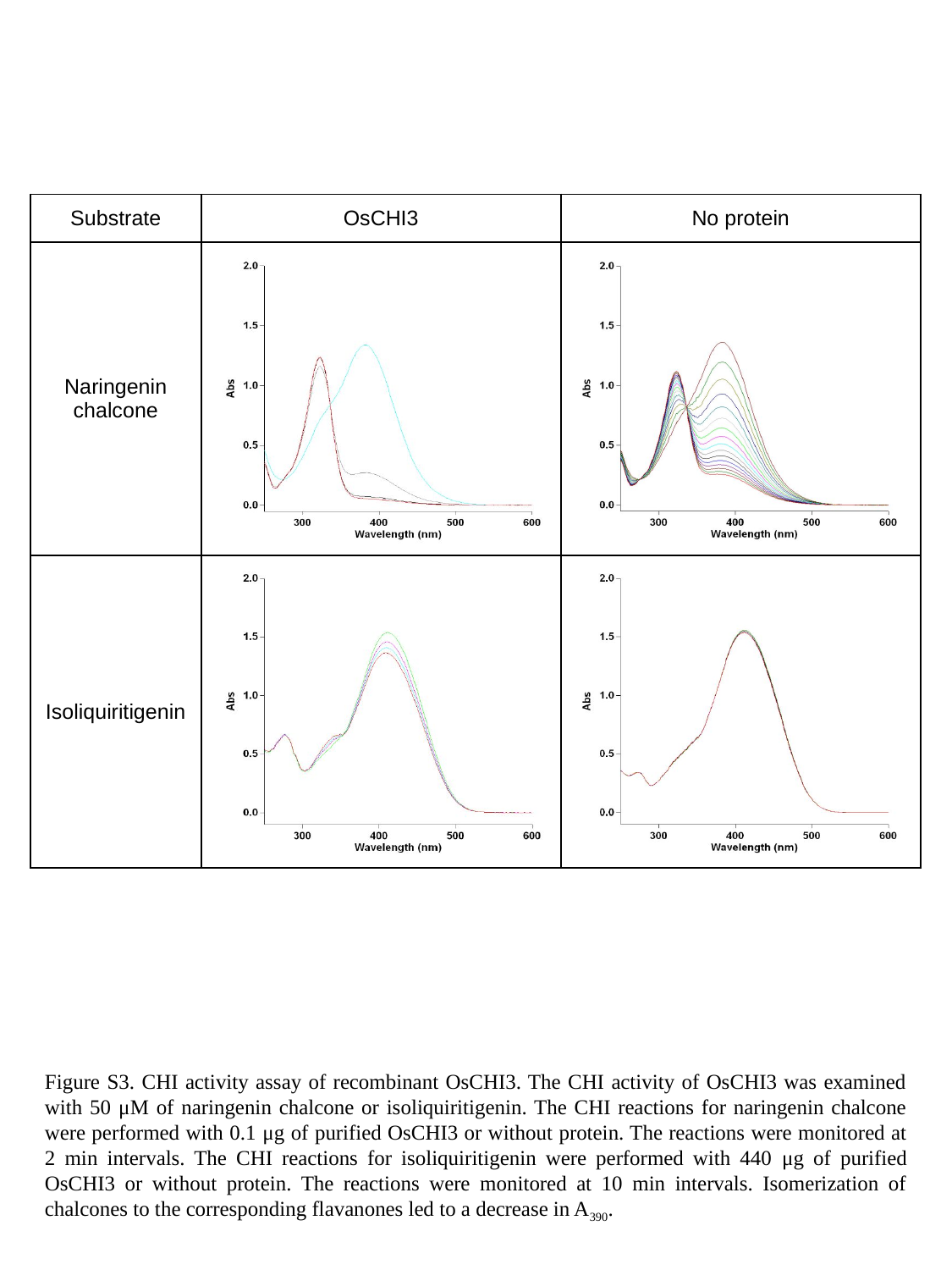

| Substrate | OsCHI3 | No protein |
| --- | --- | --- |
| Naringenin chalcone | | |
| Isoliquiritigenin | | |
Figure S3. CHI activity assay of recombinant OsCHI3. The CHI activity of OsCHI3 was examined with 50 μM of naringenin chalcone or isoliquiritigenin. The CHI reactions for naringenin chalcone were performed with 0.1 μg of purified OsCHI3 or without protein. The reactions were monitored at 2 min intervals. The CHI reactions for isoliquiritigenin were performed with 440 μg of purified OsCHI3 or without protein. The reactions were monitored at 10 min intervals. Isomerization of chalcones to the corresponding flavanones led to a decrease in A390.

## Slide 5
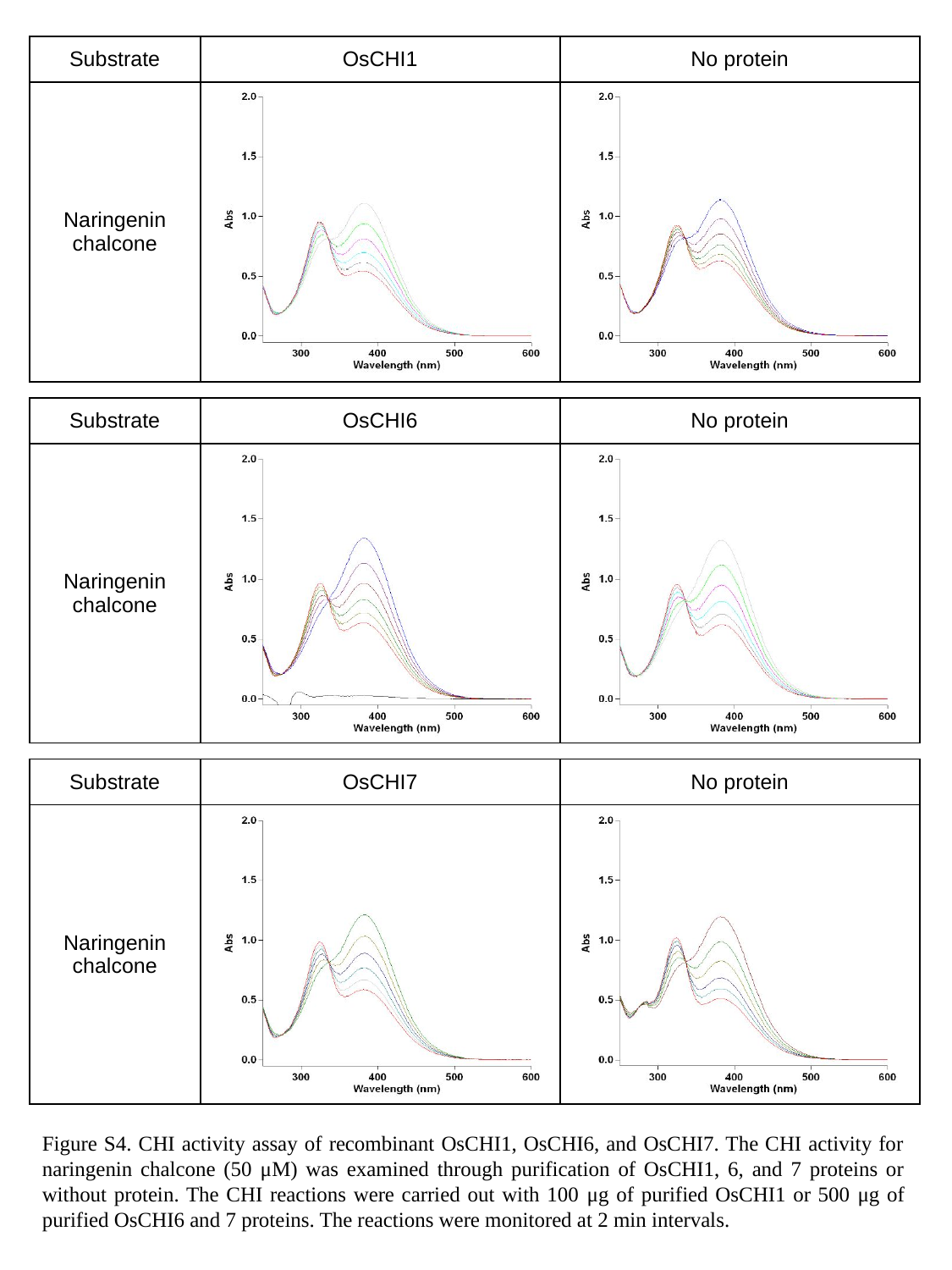

| Substrate | OsCHI1 | No protein |
| --- | --- | --- |
| Naringenin chalcone | | |
| Substrate | OsCHI6 | No protein |
| --- | --- | --- |
| Naringenin chalcone | | |
| Substrate | OsCHI7 | No protein |
| --- | --- | --- |
| Naringenin chalcone | | |
Figure S4. CHI activity assay of recombinant OsCHI1, OsCHI6, and OsCHI7. The CHI activity for naringenin chalcone (50 μM) was examined through purification of OsCHI1, 6, and 7 proteins or without protein. The CHI reactions were carried out with 100 μg of purified OsCHI1 or 500 μg of purified OsCHI6 and 7 proteins. The reactions were monitored at 2 min intervals.

## Slide 6
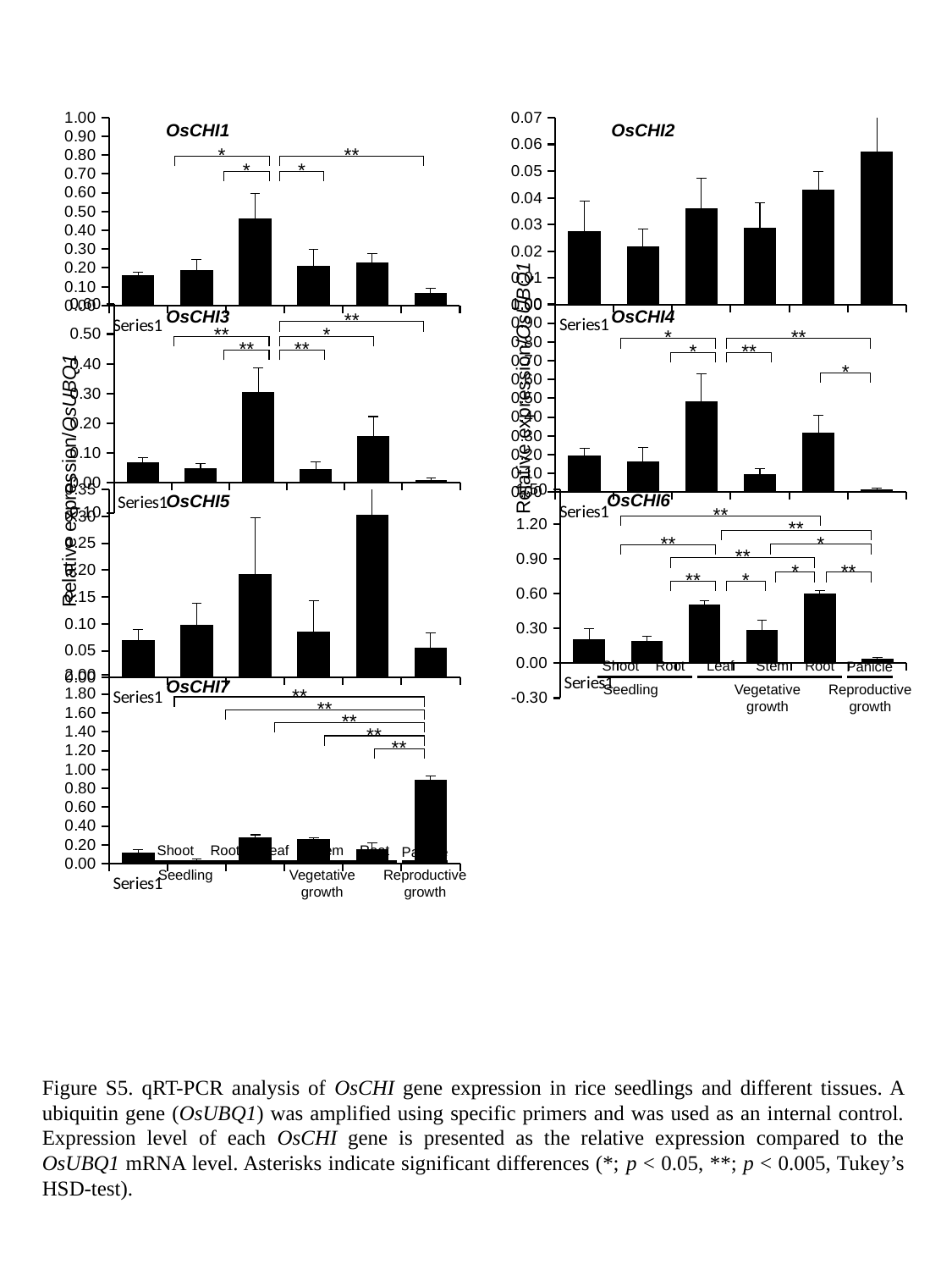

### Chart
| Category | |
|---|---|
| | 0.16047422480904155 |
| | 0.18776527449091854 |
| | 0.4630764712067414 |
| | 0.21152009765717172 |
| | 0.22963923587055762 |
| | 0.06499202339857182 |
### Chart
| Category | |
|---|---|
| | 0.027466798925695073 |
| | 0.021810764169121626 |
| | 0.03598837220386745 |
| | 0.028942434045900838 |
| | 0.042979189557419875 |
| | 0.057232739629642954 |OsCHI1
OsCHI2
*
**
*
*
Relative expression/OsUBQ1
### Chart
| Category | |
|---|---|
| | 0.1969903000230732 |
| | 0.16491737851669885 |
| | 0.4855326095496019 |
| | 0.0948573493241077 |
| | 0.31590555915228 |
| | 0.016380257066511 |
### Chart
| Category | |
|---|---|
| | 0.07036882113358595 |
| | 0.048943596713493 |
| | 0.3051268675874161 |
| | 0.04707907192250831 |
| | 0.15818781642087126 |
| | 0.010695704950474793 |OsCHI3
OsCHI4
**
Relative expression/OsUBQ1
**
*
*
**
**
**
*
**
*
### Chart
| Category | |
|---|---|
| | 0.06915721559957748 |
| | 0.0974597536349257 |
| | 0.19218739705819135 |
| | 0.08631643198239218 |
| | 0.3025731728059266 |
| | 0.05503531568923831 |
### Chart
| Category | |
|---|---|
| | 0.20789752051707508 |
| | 0.1946873736128359 |
| | 0.5061997738800464 |
| | 0.28698111255640635 |
| | 0.5992132080581611 |
| | 0.036066600025652415 |OsCHI6
OsCHI5
**
**
**
*
**
*
**
**
*
Shoot
Root
Leaf
Stem
Root
Panicle
### Chart
| Category | |
|---|---|
| | 0.11653186993301216 |
| | 0.04049563608191947 |
| | 0.27820585833298006 |
| | 0.2614841655374309 |
| | 0.15668911873334215 |
| | 0.8920275385006347 |OsCHI7
Seedling
Vegetative growth
Reproductive
growth
**
**
**
**
**
Shoot
Root
Leaf
Stem
Root
Panicle
Seedling
Vegetative growth
Reproductive
growth
Figure S5. qRT-PCR analysis of OsCHI gene expression in rice seedlings and different tissues. A ubiquitin gene (OsUBQ1) was amplified using specific primers and was used as an internal control. Expression level of each OsCHI gene is presented as the relative expression compared to the OsUBQ1 mRNA level. Asterisks indicate significant differences (*; p < 0.05, **; p < 0.005, Tukey’s HSD-test).

## Slide 7
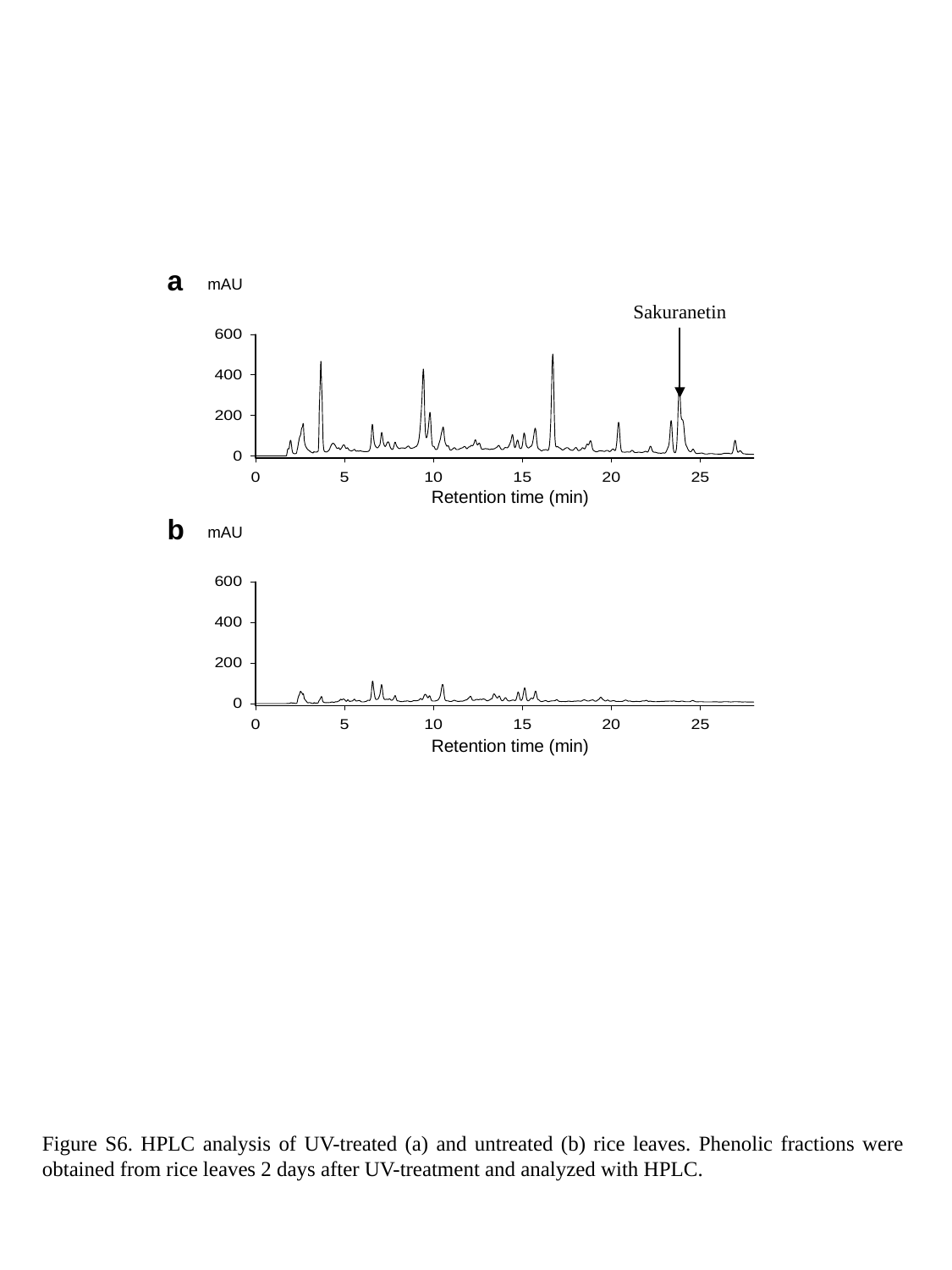

a
mAU
Sakuranetin
Retention time (min)
b
mAU
Retention time (min)
Figure S6. HPLC analysis of UV-treated (a) and untreated (b) rice leaves. Phenolic fractions were obtained from rice leaves 2 days after UV-treatment and analyzed with HPLC.

## Slide 8
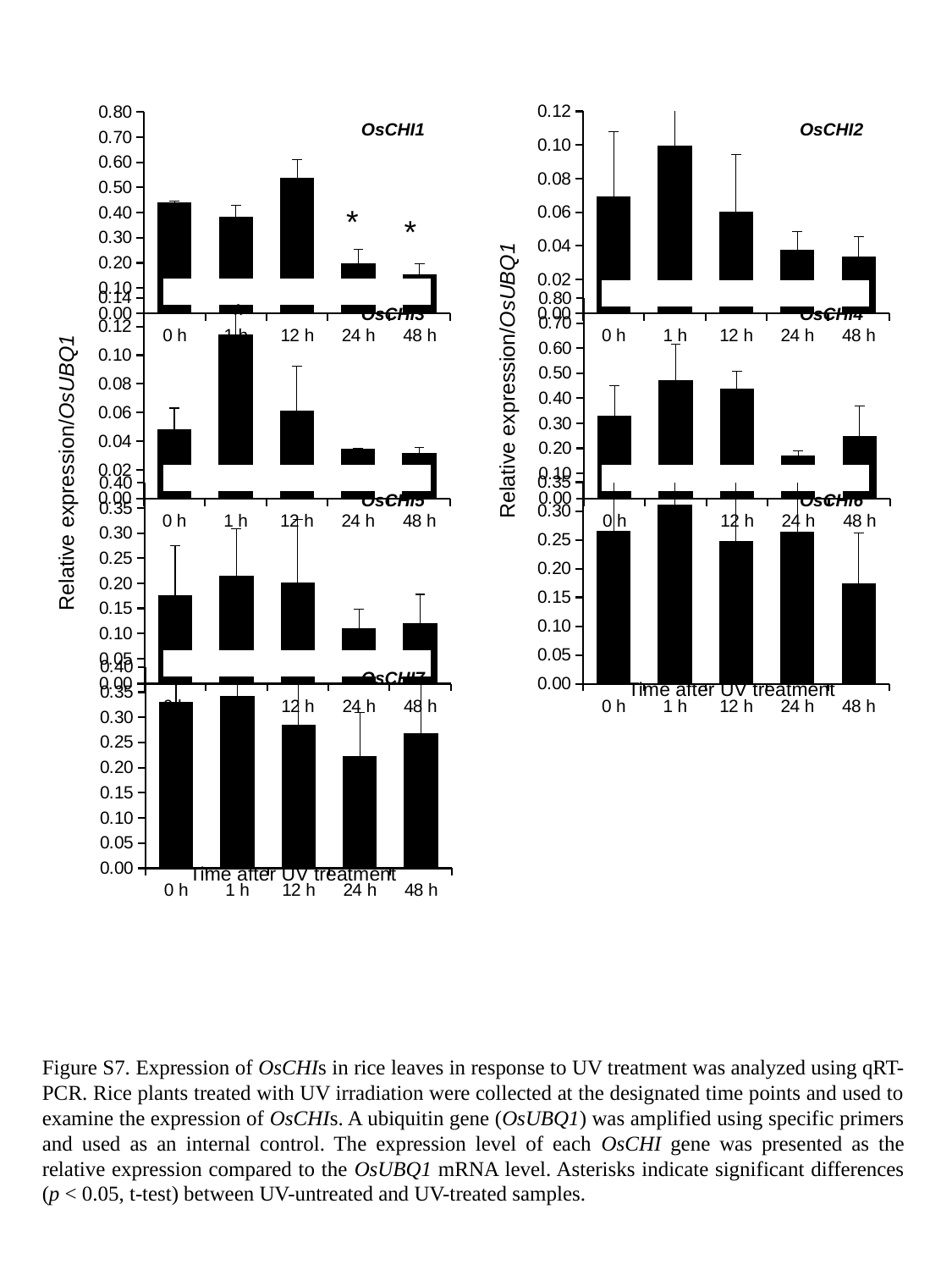

### Chart
| Category | |
|---|---|
| 0 h | 0.06941613018763038 |
| 1 h | 0.09944263160590354 |
| 12 h | 0.06050801707569944 |
| 24 h | 0.03767284577615513 |
| 48 h | 0.033668354489473425 |
### Chart
| Category | |
|---|---|
| 0 h | 0.4418491398862924 |
| 1 h | 0.38209021078570227 |
| 12 h | 0.5396419183278375 |
| 24 h | 0.19884777331438067 |
| 48 h | 0.15324409523341015 |
### Chart
| Category |
|---|OsCHI1
OsCHI2
*
*
Relative expression/OsUBQ1
### Chart
| Category | |
|---|---|
| 0 h | 0.04854636225077993 |
| 1 h | 0.11429293920969806 |
| 12 h | 0.061349168271781426 |
| 24 h | 0.03467698606579509 |
| 48 h | 0.031687674914783005 |
### Chart
| Category | |
|---|---|
| 0 h | 0.32922208165465405 |
| 1 h | 0.47239086949626663 |
| 12 h | 0.43875712762750796 |
| 24 h | 0.1726769966675102 |
| 48 h | 0.248002820967963 |*
OsCHI3
OsCHI4
Relative expression/OsUBQ1
### Chart
| Category | |
|---|---|
| 0 h | 0.26562891473517386 |
| 1 h | 0.3117221323329542 |
| 12 h | 0.24765763920156292 |
| 24 h | 0.26419994406737174 |
| 48 h | 0.17369371339859516 |
### Chart
| Category | |
|---|---|
| 0 h | 0.17647330437534145 |
| 1 h | 0.21470942222933878 |
| 12 h | 0.20228512380157357 |
| 24 h | 0.11067286017480622 |
| 48 h | 0.12035884454425196 |OsCHI5
OsCHI6
### Chart
| Category | |
|---|---|
| 0 h | 0.33068587433966273 |
| 1 h | 0.34306657982483535 |
| 12 h | 0.2846858580107428 |
| 24 h | 0.22274676377228608 |
| 48 h | 0.2692231874101586 |OsCHI7
Time after UV treatment
Time after UV treatment
Figure S7. Expression of OsCHIs in rice leaves in response to UV treatment was analyzed using qRT-PCR. Rice plants treated with UV irradiation were collected at the designated time points and used to examine the expression of OsCHIs. A ubiquitin gene (OsUBQ1) was amplified using specific primers and used as an internal control. The expression level of each OsCHI gene was presented as the relative expression compared to the OsUBQ1 mRNA level. Asterisks indicate significant differences (p < 0.05, t-test) between UV-untreated and UV-treated samples.
