# Supplementary material for: Biochemical and Molecular Characterization of the Rice Chalcone Isomerase Family
Source: Plants (Basel). 2021 Sep 30;10(10):2064. doi: 10.3390/plants10102064 (PMC8540780; doi:10.3390/plants10102064)
Supplement: Supplementary file 1 [file plants-10-02064-s001.zip › Tables S1-S3.pdf]

Table S1. Amino acid homology of OsCHIs and *bona fide* CHIs. Value represent the percentage of amino acid identity.

	DaCHI	ZmCHI	SbCHI	VvCHI	AtCHI	NtCHI	CsCHI	MsCHI	PvCHI	PlCHI	GmCHI1A
OsCHI1	11.2	11.6	11.2	12.2	12.5	12.6	14.0	13.0	12.8	13.8	12.5
OsCHI2	7.4	7.0	7.7	8.1	8.4	8.8	8.4	7.0	7.0	7.2	7.0
OsCHI3	77.8	73.6	76.5	58.9	51.4	53.6	55.6	47.2	44.0	46.4	48.9
OsCHI4	6.9	6.0	6.5	7.2	7.6	7.2	7.2	6.5	6.7	7.4	7.4
OsCHI5	14.0	14.4	15.1	13.4	13.3	13.1	15.7	13.9	14.8	14.8	13.7
OsCHI6	25.8	25.9	28.0	26.8	22.9	22.8	29.0	29.3	29.7	29.6	30.2
OsCHI7	25.6	25.7	27.8	27.8	22.5	23.5	29.3	29.4	29.5	28.7	30.4
DaCHI		77.1	81.0	59.6	52.6	54.2	57.8	45.3	43.2	44.7	46.3
ZmCHI			88.3	60.7	53.6	55.2	58.0	44.2	43.4	43.2	45.6
SbCHI				61.6	54.0	54.8	58.0	46.8	46.3	46.2	47.4
VvCHI					61.3	60.0	70.5	49.1	49.1	48.5	51.0
AtCHI						52.5	61.3	41.5	42.6	43.4	44.3
NtCHI							57.6	45.5	45.2	45.4	46.9
CsCHI								52.8	52.8	51.3	54.4
MsCHI									79.0	76.6	79.2
PvCHI										84.3	85.5
PlCHI											91.0

Table S2. Primer sequences and PCR conditions for *OsCHI* cloning. [1]

Gene	Primer sequence	Annealing Temp. (°C)
OsCHI1	5’-GGCATATGAGCGCGGTGCCCGTCGT-3’	58
(LOC_Os02g21520)	5’-CCGAATTCTCACTTTGTAAGAATAGCCT-3’	
OsCHI2	5’-CCGCTAGCATGAAACCGGACTGGTCAAT-3’	58
(LOC_Os02g53810)	5’-CCGAATTCTCAATAACATCTTCTAATGA-3’	
OsCHI3	5’-GGCATATGGCGGCCGTGTCGGAGGT-3’	56
(LOC_Os03g60509)	5’-CCGAATTCTCACGCGGACACCGGCGC-3’	
OsCHI4	5’-GGCATATGAAGGCCGATCGGTTAAT-3’	58
(LOC_Os06g10210)	5’-CCGAATTCTCATGGACAGTGATCCAAGT-3’	
OsCHI5	5’-GGCATATGGTCTCCCTCCGCTTCCC-3’	58
(LOC_Os07g38390)	5’-CCGAATTCTCAGTTCTTAAGAATAGAAG-3’	
OsCHI6	5’-GGCATATGTACTGTGCAGTGGGCAC-3’	58
(LOC_Os11g02440)	5’-CCGGATCCTCATGCAGAGAGCAGTGCTG-3’	
OsCHI7	5’-GGCATATGGCCACCGTGGAGGTGGA-3’	58
(LOC_Os12g02370)	5’-CCGGATCCTCATGCAGAGAGCAGTGCTG-3’	

[1] Restriction sites are underlined.

Table S3. Primer sequences and PCR conditions for quantitative real-time PCR analysis.

Gene	Primer sequence	Annealing Temp. (℃)
OsCHI1 (LOC_Os02g21520)	5’- TGCTGAGGAAGAACAGAGCC -3’<br>5’- TTCGAGGGTTCAAGCCAAGT -3’	58
OsCHI2 (LOC_Os02g53810)	5’- CCAACTTCAGAGGTGCTCGT -3’<br>5’- CTTTGGGCTCAGCCTTTTGC -3’	58
OsCHI3 (LOC_Os03g60509)	5’- GAGAAGTGGAAATGCCGTGC -3’<br>5’- CCGATGGCCGTGAACTTGAT -3’	58
OsCHI4 (LOC_Os06g10210)	5’- GGCAGACAAATTGGTGCTGTTA -3’<br>5’- AGTGATCCAAGTGAAGCGGG -3’	58
OsCHI5 (LOC_Os07g38390)	5’- GTGTATGCGGATGACCACGA -3’<br>5’- CATCCGTATGTCACGCTCCA -3’	58
OsCHI6 (LOC_Os11g02440)	5’- ATGTACTGTGCAGTGGGCA -3’<br>5’- GTCTGTGATGCCATTAGCAAGC -3’	58
OsCHI7 (LOC_Os12g02370)	5’- TGGACAAGTACGAGGATGAGG -3’<br>5’- TGCAATTCCAGGGGTGTTAGG -3’	58
OsUBQ5 (LOC_Os01g22490)	5’- CACCCTCGCCGACTACAACA-3’<br>5’- CTTGGTGTACGTCTTCTTCTTGC -3’	58
OsUBQ1 (LOC_Os03g13170)	5’- TGATCTGCCGCAAATGCTAT -3’<br>5’- GATTTAACCAGTCCATGAACC -3’	58
